# Market access and community size influence pastoral management of native and exotic livestock species: A case study in communities of the Cordillera Real in Bolivia's high Andean wetlands

**DOI:** 10.1371/journal.pone.0189409

**Published:** 2017-12-11

**Authors:** Quentin Struelens, Karina Gonzales Pomar, Susi Loza Herrera, Gaby Nina Huanca, Olivier Dangles, François Rebaudo

**Affiliations:** 1 UMR EGCE, UnivParisSud, CNRS, IRD, ParisSaclay, Gif-sur-Yvette, France; 2 Centro de Análisis Espacial, Instituto de Ecología, Universidad Mayor de San Andrés, La Paz, Bolivia; 3 Universidad Mayor de San Andrés, Centro de Postgrado en Ecología y Conservación, Instituto de Ecologia, La Paz, Bolivia; 4 Herbario Nacional de Bolivia, Convenio IE-MNHN, La Paz, Bolivia; 5 Facultad de Ciencias Exactas y Naturales, Pontificia Universidad Católica del Ecuador, Quito, Ecuador; Missouri Botanical Garden, UNITED STATES

## Abstract

Grazing areas management is of utmost importance in the Andean region. In the valleys of the Bolivian Cordillera Real near La Paz, pastoralism constitutes the traditional way for people to insure food security and economical sustainability. In these harsh mountains, unique and productive wetlands sustained by glacial water streams are of utmost importance for feeding cattle herds during the dry season. After the colonization by the Spanish, a shift in livestock species has been observed, with the introduction of exotic species such as cows and sheep, resulting in a different impact on pastures compared to native camelid species—llamas and alpacas. Here we explored some of the social-economical and environmental drivers that motivate Bolivian pastoralists to prefer exotic over native livestock species, based on 36 household surveys in the Cordillera Real. We constructed a Partial Least Squares Structural Equation Model in order to assess the relationships between these drivers. Our results suggest that the access to market influenced pastoralists to reshape their herd composition, by increasing the number of sheep. They also suggest that community size increased daily grazing time in pastures, therefore intensifying the grazing pressure. At a broader scale, this study highlights the effects of some social-economical and environmental drivers on mountain herding systems.

## Introduction

About 25% of the global land area is used for grazing, especially in underdeveloped countries of Africa, Asia and South America [1]. It takes place in areas where crop cultivation is impractical because of harsh conditions, and is often the only way to ensure food security [1]. In South America, 96% of llama (*Llama glama*) and alpaca (*Vicugna pacos*) herds are confined to the mountainous region of the Bolivian and Peruvian Andes [2]. Camelid herding has historically played a significant role in Bolivia’s economy [3], and about 56,000 families still depend on traditional camelid pastoralism for their livelihoods. It accounted for 0.7% of the GDP in 2002 [4], though herders are now diversifying their activities.

**Fig 1:**
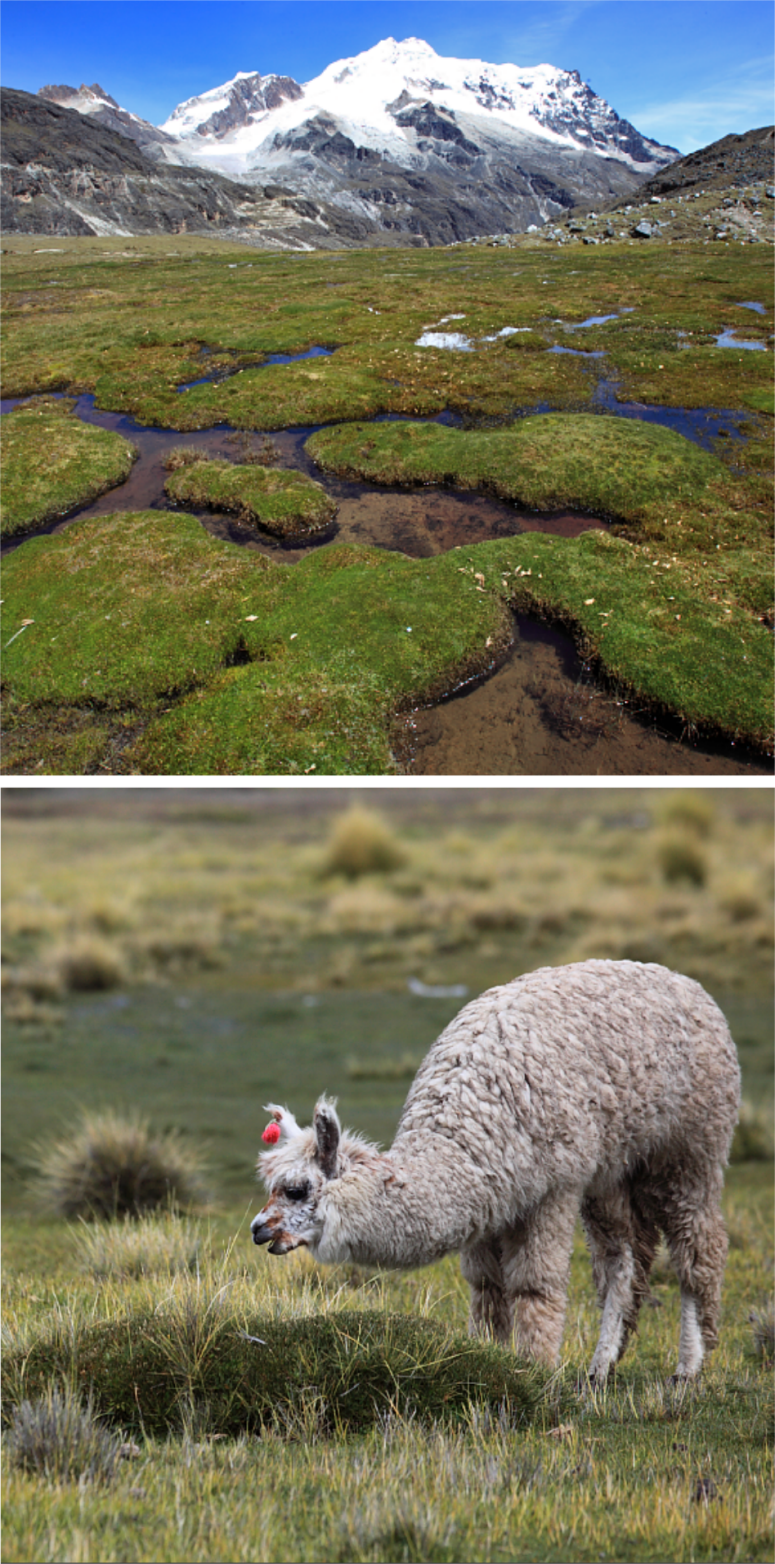
*Bofedales* at the foot of the Huayna Potosi mountain (above) and alpaca grazing on *bofedales* (below). Reprinted from http://www.biothaw.ird.fr/ under a CC BY license, with permission from Olivier Dangles, original copyright 2015.

The Bolivian Andes are mountainous (3200–6542 m) with a predominantly harsh dry climate. Solar radiation is intense during the day and temperatures dip to freezing at night (as low as -14.5°C; [5,6]). The mean temperature is 6.4°C during the rainy season, dropping to 4.5°C during the dry season [7]. The rainy season runs from December to March (average precipitation is 410.4 mm) and is marked by strong winds. The dry season runs from April to November (average precipitation is 184 mm; [5,7]). The dominant ecosystem is dry *puna* grassland, along with some wetlands (locally known as *bofedales*) that form from rain, glacial streams and ground waters flowing up to the surface [5]. These permanent pastures located in an arid terrestrial matrix sustain a unique and diverse community of organisms, ranging from microscopic aquatic species to large mammals, including native guanacos (*Llama guanicoe*) and vicugnas (*Vicugna vicugna*; [3,5,8–10]). In this landscape, domesticated camelidae (llamas and alpacas) provide local people with valuable resources, including food from the camelid meat, income from fibers, and fuel and fertilizers from manure [1–3,11] (Fig 1).

After colonization by the Spanish, exotic livestock species such as sheep and cows were introduced to the Bolivian Andes [3,12]. Mixed herds of native and non-native species are advantageous because they forage on different plants [13] and because exotic livestock meat is easier to sell to urban consumers [2]. However, unlike alpacas and llamas that are adapted to *bofedales*, sheep dig into the ground with their hooves, grasp vegetation up to the roots and consume more pasture than do camelids [12,14]. *Bofedales* deteriorate because exotic livestock destroy the vegetation and overgraze the pasture [12]. While livestock preferentially graze dry lands during the rainy period, *bofedales* are crucial for grazing during the dry season [15,16]. *Bofedales* are critical areas both for maintaining wildlife biodiversity and for sustaining human pastoral activities [2,5]. Therefore, understanding the socio-economic and environmental drivers that might encourage Andean pastoralists to prefer exotic livestock over native species is an important step towards reducing livestock pressure on *bofedales*.

Social scientists usually explore the linkages between proximate and distal drivers of human-nature interactions [17–21]. Proximate drivers are direct actions at the local scale that impact the ecosystem (e.g., grazing pressure). Distal drivers are fundamental socio-economic processes that indirectly impact the local scale through proximate drivers (e.g., access to the market or human density; [19]). However, these concepts are relative; a proximate driver can in turn be considered a distal driver relative to another variable. Studies in other parts of the world have shown that introducing pastoralists to the market economy could change their resource-use strategies by inciting some of them to shift their herd management practices to meet profit-oriented goals [22] (for a review see [23]). It is unclear how grazing pressure as a proximate driver is affected by distal socio-economic and environmental drivers.

The present study aims to understand how socio-economic and environmental drivers influence Bolivian pastoralists’ livestock quantity and preference for exotic livestock species over native camelids. We explored the relationship between three distal drivers (community size known from preliminary surveys with the leader of each community, access to market -city of El Alto, with both market for the sale of livestock and supply markets in general- and pasture extent, both recovered from satellite imagery) and five proximate drivers (alpaca quantity, llama quantity, sheep quantity, cow quantity and daily grazing duration) in Bolivian pastoral communities using Partial Least Squares Structural Equation Modeling (PLS-SEM). We hypothesized that i) easy market access (in km), and ii) a larger *bofedales* extent (in ha), would favor higher number of exotic livestock owned per household. We analyzed social data gathered from a survey conducted in four valleys of the Cordillera Real, Bolivia, where 36 households were surveyed, together with spatial environmental resource data.

## Materials and methods

### Ethics statement

Participants gave their verbal informed consent to participate to this study. The data were analyzed anonymously, and name of participants not recorded during the household surveys. No review board or ethic committee specifically approved this study, but we followed guidance from the French National Research Institute for Sustainable Development (IRD) Deontological and Ethic Committee, and the IRD good practices book for sustainable development.

### Study area

**Fig 2:**
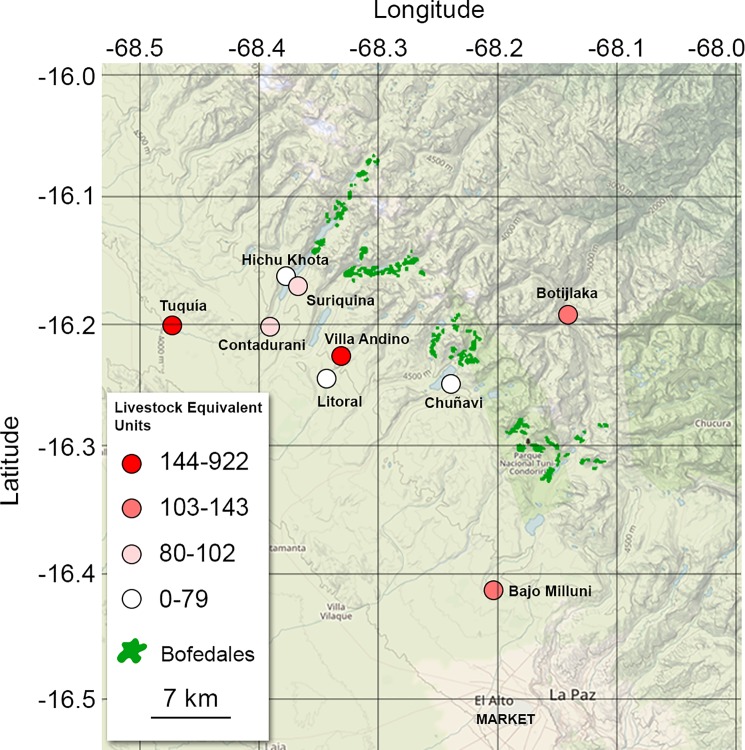
Location and livestock quantities for surveyed communities in the Cordillera Real, Bolivia. Livestock quantity from the 36 households is expressed in Livestock Equivalent Units (see text for details). Background imagery was taken from the USGS National Map Viewer (public domain).

The study was carried out in nine Aymara communities along four valleys of the Bolivian Andes in the La Paz department (16°16’S– 16°43’S latitude, 68°12’W– 68°47’W longitude; see Fig 2): Huayna Potosi (Botijlaka and Bajo Milluni communities), Tuni (Chuñavi), Palcoco (Villa Andino, Suriquina, and Litoral), Hichu Khota (Tuquia, Contadurani, and Hichu Khota Laguna), where the inhabitants have been using *bofedales* as a livestock grazing resource for millennia [15,24]. We conducted a preliminary survey that indicated that about 100 households with 600 people live in this region. It was selected for its long-standing traditions in pastoralism, its gradient of proximity to the city of El Alto, and its wide variety of herd-management strategies.

Huayna Potosí valley is located between the municipalities of El Alto and La Paz, and includes the communities of Bajo Milluni and Botijlaca, with approximately 20 households living in the valley. Principal activities in the area are structured around mining, stockbreeding, agriculture, and to minor extent tourism and jobs at the hydroelectricity plant. The Tuni Valley is located in the municipality of Pucarani, with 10 households living in the Chuñavi community with principal activities being stockbreeding, mining, and tourism. The Hichu Khota valley is located in the municipality of Batallas, with at least 400 people living in the area. The main activities are agriculture, stockbreeding, and fish farming. The Palcoco valley is located in the municipality of Pucarani, with 6 households in Litoral community and 6 households in the upper part of the valley (Suriquina and Villa Andino). The main activities are stockbreeding and fish farming. Despite the administrative division into municipalities, decisions about land use are taken at the community level, with a local leader elected for one or two years. Decisions regarding land use are first discussed in general assemblies ("tantachawi" in Aymara), so that decisions may differ from one community to another. Households in the communities apply decisions taken during general assemblies, each household being under the authority of generally one man at working age.

### Data collection

#### Survey process

We conducted structured interviews—meaning participants were asked the same questions in the same order—during the dry season between July and October 2015 [25], when grazing pressure on *bofedales* is highest. The interviews included close-ended questions used for model building and open-ended questions to set the context of the study. We sought people’s consent and informed them of how the results would be used and answered any public concerns during community meetings before the study began [26]. Participants gave their verbal informed consent to participate to this study. The data were analyzed anonymously, and name of participants not recorded during the household surveys. When conducting the interviews, we followed the guidelines described by international conventions on indigenous and tribal peoples [27] and Biological Diversity [28]. Because most people in our study region only speak Aymara, and according to Bolivian regulation (Constitución Política del Estado, 7-02-2009), interviews were conducted with the help of an Aymara-Spanish translator, who had been briefed in advance. The interview guide can be found as supporting information **S1 Text**.

We interviewed 36 households in the area that were fully completed, with answers to all questions relevant to the PLS-SEM structure (prerequisite in order to perform the PLS-SEM model analysis; 16 interviews in Hichu Khtoa, 3 in Huayna potosi, 16 in Palcoco, 1 in Tuni), and a sample representative of the variability observed in the region (36 out of 100 households). This study aims to understand how socio-economic and environmental drivers influence Bolivian pastoralists’ livestock quantity (LU) and preference for exotic livestock species over native camelids. As recommended for Structural Equation Modeling [29,30], we first defined the proximate and distal drivers to be measured on the basis of theoretical relationships (see Table 1). The drivers were assessed during the model building and those showing a high goodness of fit were kept for the analysis.

**Table 1:** Theoretical and logical justifications of the pathways included into the Partial Least Squares Structural Equation Model.

Pathway	Theoretical justification	Logical justification
*Bofedales* extent -> Community size (Number of households)	[31]	Human settlements have taken place where wetlands were larger because of the availability of water.
*Bofedales* extent -> Livestock	[32]^a)^^,^ ^b)^, [12], [14]; Sheep: [16], Llama: [33], Alpaca: [34]	Primary resources availability limits livestock population, with specificity for each livestock species.
Market access -> Community size (Number of households)	[35]^b)^	Proximity to the market attracts pastoralists that are willing to sell.
Market access -> Livestock	[36]^a)^^,^ ^b)^, [21]^a)^^,^ ^b)^	Better market access influences pastoralists to become market-oriented.
Community size (Number of households) -> Livestock	[37–39]^a)^	Human resources living in the community decide of the quantity of livestock (LU) that is manageable (llamas and alpacas need more grazing time; different foraging niches and bite rates between livestock species; llamas are better adapted to drier regions).
Community size (Number of households) -> Grazing duration	[40]^a)^^,^ ^b)^	Tragedy of commons concept: The size of the group using the common-pool resource influences the collective use of this resource, as decisions regarding collective use are decided locally.

^a)^ Study from another area.

^b)^ Study about another similar driver.

#### Proximate drivers

The proximate drivers included in model construction were i) the number of cows and sheep owned per household, ii) the number of llamas and alpacas owned per household and iii) the daily duration that livestock grazes in bofedales, based on the respondent answers on a weekly basis. Native livestock was separated from exotic livestock as grazing effects on vegetation are known to differ among species (e.g., for goat and llamas [41]). The grazing duration represents dependence and access to grazing areas and water bodies. Livestock quantity was transformed into Livestock Equivalent Units (LU) to account for differences in individual grazing capacity across species. We based our equivalences on the metabolic weight (i.e., body weight^0.75^) obtained from the measured average weight of the animals in the study area [42]. Because llamas are the most common animal species grazing on *bofedales*, we used its average weight as the reference for LU calculations (1 llama = 72 kg = 1 LU; 1 alpaca = 0.805 LU; 1 sheep = 0.444 LU; 1 cow = 3.89 LU).

#### Distal drivers

The economic distal driver was related to market access, and was assessed as the distance by road to the closest city [21,36]: in this case, El Alto, with nearly one million inhabitants. Market access was considered as a distal driver because distance is a proxy for the time needed to reach the city, which may vary among communities depending on vehicle unpredictable availability. Market access was linked to community size because proximity to a market is attractive for pastoralists that are willing to sell. Moreover, market access was related to livestock quantity (in LU) because proximity to market influences livestock quantity and herd composition (Table 1). Social distal driver accounted for the number of households in the community, which has been shown to influence livestock quantity in a previous study from Peru and Bolivia [2]. Household number in the community was considered as a distal driver because some people are living in the city but spend most of the time in the community, and *vice versa*. This social driver was related to livestock quantity and grazing duration in *bofedales* because it influences the manageable livestock quantity and collective decisions about resources use, respectively (Table 1). Moreover, the available human resources to manage livestock were highlighted by many interviewees as key for a sustainable pastoralism activity. Environmental distal drivers, such as the absolute area of *bofedales* (total grazing pasture) and relative extent (relative to the area of the valley) for each valley were included in the analysis [2] (see [10]), because *bofedales* are limiting factors during the dry season when no other grazing areas are available. During the dry season, *bofedales* represent a key resource as water and grazing areas, whose quantity and quality depend on the *bofedales* extent, and availability on the valley extent. The *bofedales* extent was linked to community size [10], and to livestock quantity because it represents the resources animals and pastoralists depend on (Table 1). Economic and social drivers were collected through structured interviews, and environmental drivers related to *bofedales* extent were gathered using satellite imagery and processed using the Geographic Information System (GIS) software ArcGIS (ESRI, Redlands, CA, USA) in a previous study [10]. The extent of *bofedales* ecosystems by valley was determined visually from 2013 imagery, and checked manually in the corresponding areas using a GPS, as described by [10].

### Model building

To explore the relationship between distal and proximate drivers, we constructed a Structural Equation Model (SEM; [30]). SEM relies on a combination of regression techniques, path analysis and confirmatory factor analysis to identify complex causal patterns between proximate and distal drivers, based on theoretically designed hypothesis [19,30,43]. SEM techniques are widespread in socio-economic studies because they can be used to compute abstract drivers (latent drivers) from observed drivers and to quantify causal effects between drivers [30,43]. SEMs can be broken down into two sub-models: a measurement model and a structural model. The measurement model depicts the construction of latent drivers from indicator variables (i.e., measured). The structural model draws the causal relationships between previously constructed latent drivers [30,43].

Among SEM, two options are commonly used for path estimation: the first relies on covariance-based methods, and the second on Partial Least Squares (PLS) methods. Covariance-based methods are best suited for theory testing whereas PLS methods are best suited for exploration. The latter are also more robust when relying on small sample sizes and do not assume a normal data distribution [29,44,45]. Because of the characteristics of our sample size, we chose PLS over covariance-based methods. In the case of PLS-SEM, the two sub-models are evaluated independently [30,45,46]. Another specificity of PLS-SEM is that they are exclusively recursive models, which implies that rank and order conditions do not have to be met to identify a model [30,47]. The loadings and unidimensionality of measurement models characterize their ability to reflect their indicator. Loadings are the weightings that reflect the correlation between the latent driver and its indicator variables. Unidimensionality is a measure of internal consistency that reflects how closely related the indicator variables of a latent driver are to one another [45,46]. We ensured unidimensionality in the model by selecting latent variable constructs that showed both a Cronbach’s Alpha and a Dillon-Goldstein’s rho greater than 0.7 [30,45]. We also ensured that measurement constructs' loadings were higher than 0.7, and that the cross-loadings were lower than the loadings [30,45]. When cross-loadings are higher than loadings, it means that some indicator variable better explains a different latent driver than the one it belongs to. To assess the quality of the structural model, r-squared values for endogenous variables indicate the variation explained by latent constructs. The data were standardized to establish hierarchies between drivers. Statistical analyses were performed using R v3.2.5 [48], and the PLS-SEM was performed using the *plspm* R package [30].

**Fig 3:**
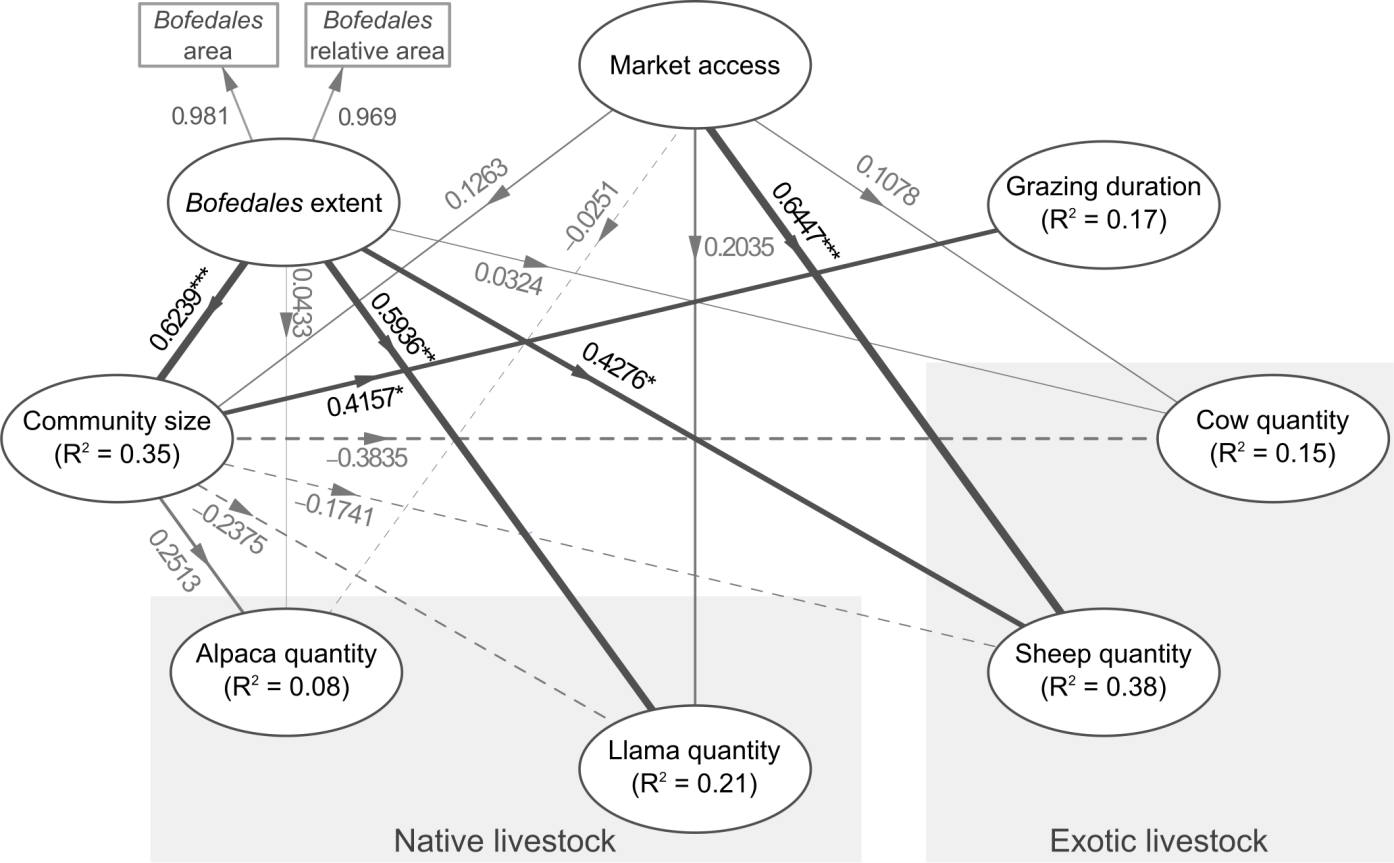
Structural model of the Partial Least Squares Structural Equation Model showing most probable relationships between latent drivers (ellipses) constructed from indicator variables (rectangles). R-squared values for endogenous latent variables show the amount of variation explained. Negative path weights are shown as dashed lines, positive correlations as plain lines. Line widths are proportional to the path weights. * p-value < 0.05; ** p-value < 0.01; *** p-value < 0.005. Note that indicators are not shown for latent drivers constructed from one indicator variable only.

The structural and measurement models of our hypothesis are shown in Fig 3, where all drivers are observed variables except for *bofedales* extent, which is a latent variable derived from relative and absolute *bofedales* extents. We justified the pathways between drivers based on previously established results in the literature and logical assertions (Table 1; [30,49]). These hypothesized relationships were selected from a prior analysis of one-to-one Pearson correlations, as recommended for PLS-SEM [30] (see S1 Fig). Differences between exploratory analysis and final PLS-SEM topology are due to the number of unanswered questions that reduced the sample size to be used in the PLS-SEM.

## Results and discussion

From the 36 interviews, total livestock varied between zero livestock equivalent units (LU) and 143 LU per household and between 20 LU and 922 LU per community (Table 2 and Fig 2). Native livestock varied between zero LU and 100 LU per household, whereas exotic livestock varied between zero LU and 128.77 LU per household (Table 2; t = -0.548, df = 67.911, p-value = 0.585). Alpacas varied between zero and 48.33 LU per household, llamas between zero and 70 LU per household, sheep between zero and 88.89 LU per household, and cows between zero and 46.67 LU per household. The proportion of native livestock varied between zero and 100% of herd composition per household. Distance to the market in El Alto ranged from 5.7 km to 60 km. The *bofedales* areas varied between 55.71 ha and 84.33 ha, and the relative area of *bofedales* per valley ranged from 0.42% to 2.67%. The number of households per community (households defined under the authority of a local leader) varied between 2 and 17. Finally, the daily grazing time in *bofedales* varied between 10 min/day and 10 hours/day, revealing different grazing strategies among households in our study area.

**Table 2:** Summary of the indicators variables used in the Structural Equation Modeling and how they were collected. Ha = hectares, km = kilometers, LU = Livestock Equivalent Units (1 llama = 72 kg = 1 LU; 1 alpaca = 0.805 LU; 1 sheep = 0.444 LU; 1 cow = 3.89 LU), min = minutes and d = day, GIS = Geographic Information System, sd = standard deviation, based on the 36 interviews analyzed. Range is followed by average value per valley (Hichu Khota, Huayna Potosi, Palcoco, Tuni, respectively).

Indicator variable	Justification	Source	Range (mean/valley)	Mean (sd)
Distance to El Alto (km)	Market access variable	GIS data	5.70–60.00; (51; 23; 50; 51)	47.50 (8.70)
*Bofedales* area (ha)	Availability of grazing areas	GIS data	55.71–84.33; (77.8; 80.9; 55.7; 84.33)	68.44 (11.62)
Relative *bofedales* area per valley (%)	Proportion of grazing areas available during the dry season	GIS data	0.42–2.67; (1.15; 0.42; 2.61; 1.20)	1.80 (0.85)
Number of households/community	Grazing areas management	Social survey	2–17; (8.2; 3.8; 8.3; 6.0)	12.31 (5.01)
Native livestock (LU/household)	-	Social survey	0–100; (20; 17; 34; 64)	26.25 (21.66)
Exotic livestock (LU/household)	-	Social survey	0–127.78; (29; 32; 42; 16)	27.92 (25.85)
Alpacas (LU/household)	-	Social survey	0–48.33; (5; 0; 6; 10)	6.13 (12.44)
Llamas (LU/household)	-	Social survey	0–70; (15; 17; 28; 55)	18.53 (21.19)
Sheep (LU/household)	-	Social survey	0–88.89; (11; 8; 23; 9)	15.17 (19.46)
Cows (LU/household)	-	Social survey	0–46.67; (18; 23; 18; 7)	12.75 (14.09)
Daily grazing duration in *bofedales* (min/d)	Dependence and access to grazing areas	Social survey	10–600; (298; 80; 406; 120)	330.3 (208.02)

Our model showed an overall goodness-of-fit of 0.46, resulting from the endogenous variables’ r-squared values of 0.17 for grazing time, 0.15 for cow quantity (LU), 0.38 for sheep quantity (LU), 0.21 for llama quantity (LU), 0.008 for alpaca quantity (LU), and 0.35 for community size (Fig 3).

Our results suggest causal relationship for five out of fifteen relationships between latent drivers (Fig 3). *Bofedales* extent showed a positive effect on llama (path-weight = 0.21) and sheep quantities (path-weight = 0.38). *Bofedales* extent also suggests causal effect on community size (path-weight = 0.62). Community size in turn showed an effect on the daily grazing duration in *bofedales* (path-weight = 0.42). Finally, the access to market showed a positive effect on sheep quantity (LU; path-weight = 0.65), but no effect on other livestock species quantities. No relationship was found for cow and alpaca quantities per household. In the case of alpaca, this lack of significant relationship could be due to its rarity in the sample.

This study aimed to understand how distal socio-economic and environmental drivers determine the abundance and species choices of native and non-native livestock in *bofedales* close to El Alto, Bolivia. Our results suggest that households closer to market had proportionally more sheep (exotic species) than other livestock species, supporting previous studies [22]. We also suggest that pastoralist households located in a valley with a greater extent of *bofedales* tended to have more sheep and llamas. Finally, our results tended to show that the size of the community, which was influenced by the extent of *bofedales*, was the main driver of the daily grazing time in *bofedales*. Note that we were not able to verify during the interviews the causalities determined by the model.

### Explaining the effects of distal drivers on livestock quantity and grazing duration

#### Proximity to the market increases sheep quantity (LU)

Proximity to the market, as an indicator of market access, has been shown to influence human natural resource use in Africa and Oceania [21,36,50]. This is particularly true in areas where people have traditional practices of natural resource use; market access has the power to change habits, often resulting in ecosystem deterioration [22,51,52]. Strong traditional knowledge guides habits in pastoral communities in Bolivia and around the world, and these habits are therefore sensitive to socio-economic change [15,23,53].

Globally, modernization, including access to national and global markets, is influencing pastoralists [23]. [22] showed that expanded market access drove some Kenyan pastoralists to reshape their herd-management strategies in response to market competitiveness, which resulted in common pasture degradation. Similarly, in Bolivia and Peru, [2] observed that herders that are close to markets tend to become market-oriented, which is consistent with our results: households closer to the market tended to have a mixed herd with a greater proportion of sheep (Fig 3; [2]). However, even though bofedales are ecosystems scattered over most of the Southern Andean, the Aymara cultural context in Bolivia may not make the observed results generalizable across the region. Also, herders that are farther from the market also live higher in the valleys; therefore, the relationship between market access and exotic livestock quantity could also be explained by the effect of high altitude on exotic livestock. Indeed, previous studies showed that mortality from pulmonary edema is higher in exotic livestock such as sheep at higher altitudes [3,11]. Nevertheless, in our study ranging from 3500 to 4600 m.a.s.l., we found no significant correlation between the number of exotic livestock and elevation (Pearson correlation: r = -0.073; t = -0.426, df = 34, p-value = 0.673). The household surveys used in this study did not allow determining which part of the livestock was exclusively market-oriented and which part was for auto-consumption or local trade between farmers. If no farmer in these areas is exclusively oriented toward the market, that information could have raised more evidence on the impact of market proximity on exotic livestock quantity. In this analysis, we considered the distance in km to the nearest market. It is likely that “time” may be a better variable than “distance” to characterize farmers’ remoteness to market. However, travel time depends on poorly predictable events of transport units stopping by farmers’ house, which may greatly influence their decision to travel. Bolivia is one of the poorest countries in South America and transportation is generally strongly constrained by demand (i.e., there is no transportation if not enough people to transport). This information was unfortunately not available for our study. These results should also be contrasted by other economic and productive activities in the region, and in particular mining and agriculture, which influence the time dedicated to livestock and reduce the importance of the activity, even if we observed that pastoralism remains the main activity.

#### Community size increases grazing time

*Bofedales* pastures are subtractable, common-pool resources, meaning that when one member of the community uses the resource, there is less remaining for others [54]. Resources are subject to depletion, degradation or overuse (overgrazing in the case of *bofedales* pastures). However, Bolivian pastoralists respect strict rules imposed by the traditional organization of communities (*Ayllu*) that are designed to balance the pasture, livestock population and human population [53,55]. But since the agrarian reform (1953), more and more traditional *Ayllu* land management has been replaced by private land ownership [53]. Moreover, the opening to the market may drive some pastoralists to follow a self-interested economic goal, which could lead to resource overuse [21,22]. In common-pool resource management in forests, group size has been negatively correlated with collective actions [40]. Smaller groups foster greater trust between members, which creates favorable conditions for collective actions [40]. Our findings may be consistent, as they showed that herds from larger communities tended to spend more time in the *bofedales* pastures, potentially leading to overuse (Fig 3). However, we also found that the community size was influenced by the extent of available pasture, which could support longer grazing periods and sustain a larger population of livestock and human. Our model is unable to disentangle these opposing drivers.

#### *Bofedales* extent increases llama and sheep quantity (LU)

Previous studies showed that alpacas prefer grazing on wet vegetation such as *bofedales*, whereas llamas are less selective and can graze on dryer pastures [55,56]. Here, we report that *bofedales’* extent was directly correlated to llama population size but not alpaca's (Fig 3). A study in a similar environment in Chile showed a general overlap of diet [57]. These contradictory results highlight the fact that camelid diet is highly adaptive and may vary between regions and climates [58]. [2] found that *bofedales* extent was a main driver of livestock quantity among Bolivian and Peruvian pastoralists. Our study goes further and suggests that the bofedales extent principally affects llamas and sheep, and has a small and non-significant effect on cows and alpacas (Fig 3). However, other factors not analyzed in this work, such as the availability of grazing areas outside of bofedales or the distance to the grazing areas, may also be important to fully appreciate the relationship between bofedales extent and cattle type.

#### Study limitations

Climate change has had a marked impact on the study region [7,59]. However, climate change variables were not used in our PLS-SEM analysis, as the current perception by most farmers is that “*bofedales will stand for ever*”. This perception can be explained by the fact that, over the last decades, trends in *bofedales’* cover in the Royal Cordillera have been complex. While some *bofedales* have suffered increasing rates of drying and fragmentation others have increased in area as a result of glacier melting [60]. Climate change is likely to have important consequences of *bofedales’* pastoralism, yet these are not fully perceived by local people.

Decision-making remains a complex process involving many factors, and our PLS-SEM analyses, although testing our hypotheses on the main drivers of grazing pressure, ignore the implicit multi-factorial complexity of social-ecological systems [61]. The analytical design of PLS-SEM (and SEM in general) is indeed a simplified representation of the real processes at stake, balancing the model complexity with the ability to interpret the model output results, similar to the model complexity consideration in agent-based modeling [62]. For example, simplified representation approaches have been successful to represent resources consumption by herders in Sahel region using an agent-based model [63], or pathways explaining resilience of pastoralist systems in Ethiopia using SEM [64]. In the case of Andean bofedales, this study is a first step towards understanding the role of socio-economic drivers on grazing pressure in the Andes. The next steps would be to integrate more economic (e.g. meat market price, bofedales property rules, supplementary activities and revenues), social (family composition and activities, bofedales management), cultural (e.g., wetland perception and traditions), or environmental factors (e.g. rainfall, number of freezing days) [65] (see S1 Fig for other factors not included in this analysis due to the limited number of interviews). Moreover, the different levels of remoteness and natural resources of each valley may play a role in pastoral management. While this is partially taken into account through market access, a further step would need investigating the social processes at play in wetlands’ management and their impacts on pastoralism-related decisions. Note that because the study was carried out during the dry season, when grazing pressure on *bofedales* is the highest, the results may not be transferable to the rainy season.

## Conclusion

Based on a limited number of interviews (inherent property of the system despite representativeness of the communities living in the study area), our study lacks the statistical requirements to perform a SEM analysis. That is why we used the PLS-SEM analysis, a non-parametric method validating our model. Our study underlines several main drivers of herd management and subsequent grazing pressure in the Cordillera Real: access to the market drives the proportion of sheep in a herd, community size drives daily grazing time in *bofedales*, and *bofedales* extent drives the number of llama and sheep. By using PLS-SEM, we have been able to disentangle the effects of socio-economic and environmental drivers on various components of grazing pressure. Balancing wetland conservation with sustainability for pastoralists relies on incentives that increase the value of native species. It is unlikely that pastoralists in our study area will stay away from the market, given their proximity to a rapidly growing urban centre. Therefore, herds will likely become bigger with fewer collective *bofedales* use patterns. Nevertheless, the pressure on *bofedales* could be reduced if lower-impact native livestock species are favored over sheep. Even if many possible events may affect *bofedales* leaving many aspects open in this study, this could be achieved by improving infrastructure for safe llama meat processing, which herders still mainly use for their own consumption [2,3]. Our study also suggests that smaller communities are more likely to promote collective action and management of common resources such as *bofedales*. Therefore, another way to reduce grazing pressure could be promoting smaller communities. At a broader scale, and considering the decline in pastoral communities across the globe [23], this study highlights the benefits of using PLS-SEM to identify most likely causal relationships between drivers, and propose management plans that act at the root of key issues.

## Supporting information

S1 Fig Schema summarizing exploratory results of one-to-one correlations between environmental, economic and social predictors. r = Pearson product-moment correlations; p = p-value of the Pearson correlation test. Plain arrows represent positive correlations and dashed arrows stand for negative correlations.
(PDF)

S1 File Data used for Fig 2 in CSV format. In the CSV file, "comunidad" refers to the community, "totalGanado" to the total number of animals in Livestock Equivalent Units, "lon" to longitude, and "lat" to latitude.
(CSV)

S2 File Data used for Fig 3 in CSV format. In the CSV file, "distAlto" refers to the market access, "extbof" to the bofedales area, "extbofRel" to the bofedales relative area, "dens" to the community size, "alpaca" to the alpaca quantity in LU, "llama" to the llama quantity in LU, "ovino" to the sheep quantity in LU, "bovino" to the cow quantity in LU, and "tps" to the grazing duration.
(CSV)

S3 File Data used for S1 Fig in CSV format. In the CSV file, "otra_occ_madre" refers to the mother supplementary activity (Boolean), "otra_occ_padre" to the father supplementary activity (Boolean), "otra_occ_ambos" to either father or mother supplementary activity (Boolean), "dist_from_road" to the distance to the closest road (km), "tiempo_viviendo_sitio_madre" to the time lived by the mother in community (years), "tiempo_viviendo_sitio_padre" to the time lived by the father in the community (years), "tiempo_viviendo_sitio_madre_padre" to the average time lived either by the mother or the father in the community (years), "propiedad_bofedal" to the bofedal property (factor), "households_per_community" to the community size, "ganado_llamas" to the number of llama heads, "ganado_alpacas" to the number of alpaca heads, "ganado_bovino" to the number of cow heads, "ganado_ovino" to the number of sheep heads, "ganado_total" to the number of animal heads in LU, "num_casas" to the number of houses own by the household, "vende_produccion" to the production destination (factor), "relative_extent_bofedal" to the relative bofedales extent, and "dist_from_bofedal" to the distance to the closest bofedal in km.
(CSV)

S1 Text Copy of the interview guide used in the study, in both the original language and English. (DOCX)
