# Supplementary figures and images for: Market access and community size influence pastoral management of native and exotic livestock species: A case study in communities of the Cordillera Real in Bolivia's high Andean wetlands
Source: PLoS One. 2017 Dec 11;12(12):e0189409. doi: 10.1371/journal.pone.0189409 (PMC5724826; doi:10.1371/journal.pone.0189409)

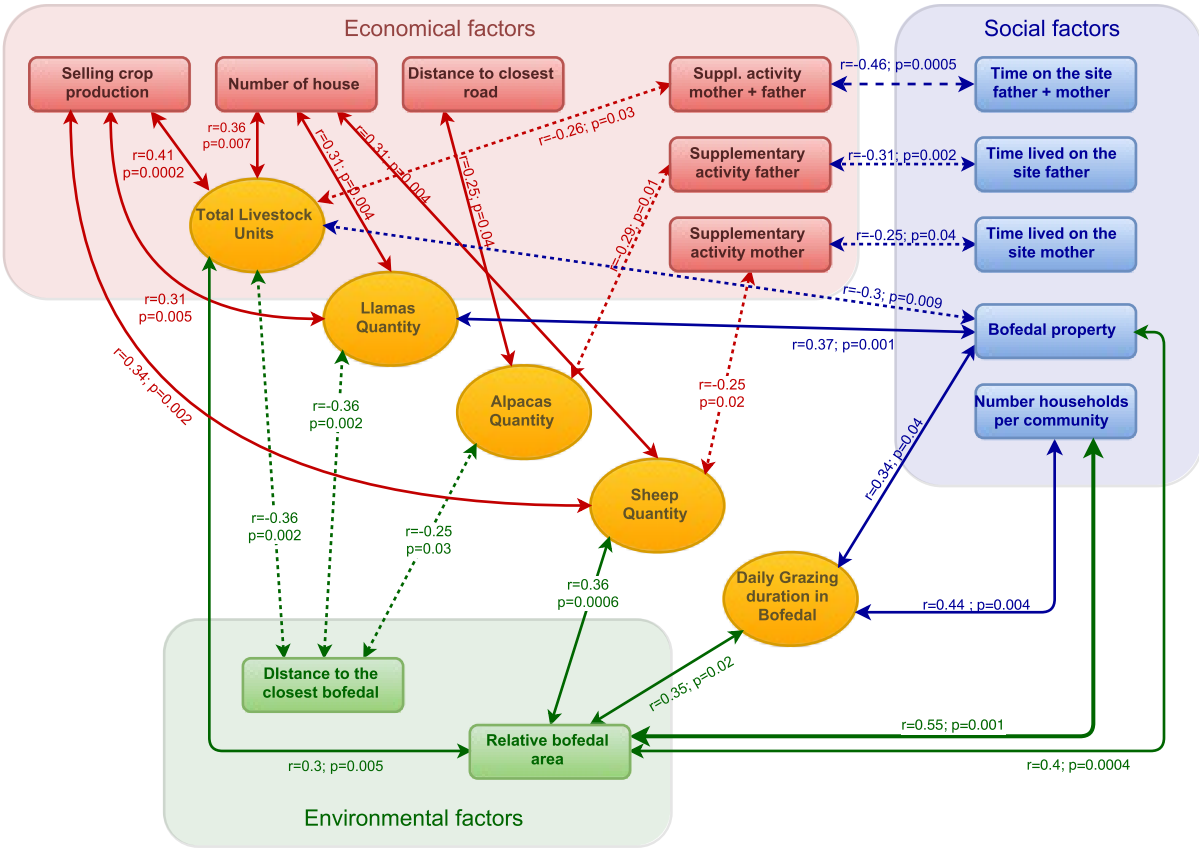

Supplement: S1 Fig — r = Pearson product-moment correlations; p = p-value of the Pearson correlation test. Plain arrows represent positive correlations and dashed arrows stand for negative correlations. (PDF) [file pone.0189409.s001.pdf]
