# Supplementary material for: Market access and community size influence pastoral management of native and exotic livestock species: A case study in communities of the Cordillera Real in Bolivia's high Andean wetlands
Source: PLoS One. 2017 Dec 11;12(12):e0189409. doi: 10.1371/journal.pone.0189409 (PMC5724826; doi:10.1371/journal.pone.0189409)
Supplement: S1 Text — (DOCX) [file pone.0189409.s005.docx]

**Supporting Information S2:** **copy of the interview guide used in the study**, in both the original language and English

| **Spanish** | **English** |
| --- | --- |
| ID | ID |
| Valle | Valley |
| Comunidad | Community |
| **Características de las personas y la familia** | **Household characteristics** |
| Edad papa | Age head of household (HH) |
| Edad mama | Age spouse head of household (SHH) |
| Lugar de nacimiento papa | Head of household place of birth |
| Lugar de nacimiento mama | Spouse head of household place of birth |
| Tiempo viviendo en el lugar papa | Time living in the area HH |
| Tiempo viviendo en el lugar mama | Time living in the area SHH |
| Grado educación papa | Education level HH |
| Grado educación mama | Education level SHH |
| Tamaño familia | Number of people in the family |
| Familia (abuelos) vivían aquí | Grandparents living here |
| Tiene otras casas fuera de la comunidad | House outside the community |
| Por que | Why |
| Donde | Where |
| Tiene vehículos | Vehicle |
| Tiene luz | Electricity at home |
| Tiene agua potable | Drinkable water at home |
| Tiene alcantarillado | Sewage system |
| **Observación adicional** | **Observations** |
| Nº de habitaciones (construcciones) | Number of rooms |
| Material de construcción de la casa | Material for the house |
| Hay corrales | Pen |
| Materiales de construcción del corral | Material for the pen |
| Existe camino para movilidad a su casa | Path suitable for motor vehicles |
| **Actividades económicas - productivas de la familia** | **Familial economic and productive activities** |
| Qué actividades realizan papa | HH activities |
| Qué actividades realiza mama | SHH activities |
| Qué actividades realizan los hijos | Children activities |
| Quiénes realizan actividades pagadas | Paid activities |
| En que | Which |
| Donde | Where |
| Cuánto tiempo | How much time |
| En que meses | Which months |
| Cuál es la actividad principal de la familia | Principal activity |
| Qué ganados tiene | Livestock |
| Cuál es su ganado principal | Principal livestock |
| Porque | Why |
| Dónde lleva a pastear | Grazing areas |
| Cuánto ganado tiene | Number of livestock heads |
| Cuántas hectáreas ocupa para pastear | Number of hectares |
| Cuánto horas pastea en el bofedal | Grazing time |
| Tiene cultivos | Cultures |
| Donde | Where |
| Cuándo siembra | Sowing period |
| Cuándo cosecha | Harvest period |
| Dónde lleva a vender | Market place to sell |
| Quién se encarga de vender | Person in charge of selling |
| Considera que los jóvenes tienen futuro en el campo | Do children have a future in the countryside |
| Cuál era su actividad principal hace 5 años | Principal activity 5 years ago |
| **Características del manejo de bofedal** | **Wetland management characteristics** |
| Hacen el manejo del bofedal | Wetland management |
| Como | How |
| Como se organizan para el uso de los bofedales | Organization |
| Cuales son los meses del año en que usan los bofedales | Months where wetlands are used |
| Por que | Why |
| Cuánto ganado pastean por hectárea | Number of livestock heads per hectare |
| Separan el ganado para pastear | Separation of livestock for grazing |
| Como | How |
| Cuál es la distancia del corral hacia los bofedales | Distance from pen to wetlands |
| Hay mejores lugares para pastear en época seca | Existence of grazing areas in arid period |
| Donde | Where |
| Tienen lugares donde no debe entrar el ganado | Prohibited areas |
| Por que | Why |
| Si es por el sistema de rotación pastoril por cuánto tiempo | If for rotation purposes, how much time |
| **Percepciones del cambio** | **Change perception** |
| Son importantes los bofedales para usted | Wetland importance |
| Porque | Why |
| Qué sucedería si no existieran los bofedales | What would happen if no existence of wetlands |
| Se está haciendo algo para solucionar estos problemas | What can be done to solve these problems |
| Qué se hace | What is done |
